# An Automatic Domain-General Error Signal Is Shared across Tasks and Predicts Confidence in Different Sensory Modalities

**DOI:** 10.1523/ENEURO.0124-25.2025

**Published:** 2025-06-05

**Authors:** Matthew J. Davidson, Sriraj Aiyer, Nick Yeung

**Affiliations:** ^1^Discipline of Psychology, University of Technology Sydney, Sydney, New South Wales 2008, Australia; ^2^Department of Experimental Psychology, University of Oxford, Oxford OX2 6NW, United Kingdom

**Keywords:** audition, confidence, decision-making, EEG, metacognition, vision

## Abstract

Understanding the ability to self-evaluate decisions is an active area of research. This research has primarily focused on the neural correlates of self-evaluation during visual tasks and whether neural correlates before or after the primary decision contribute to self-reported confidence. This focus has been useful, yet the reliance on subjective confidence reports may confound our understanding of key everyday features of metacognitive self-evaluation: that decisions must be rapidly evaluated without explicit feedback and unfold in a multisensory world. These considerations led us to hypothesize that an automatic domain-general metacognitive signal may be shared between sensory modalities, which we tested in the present study with multivariate decoding of electroencephalographic (EEG) data. Participants (*N* = 21, 12 female) first performed a visual task with no request for self-evaluations of performance, prior to an auditory task that included rating decision confidence on each trial. A multivariate classifier trained to predict errors in the speeded visual task generalized to distinguish correct and error trials in the subsequent nonspeeded auditory discrimination. This generalization did not occur for classifiers trained on the visual stimulus-locked data and further predicted subjective confidence on the subsequent auditory task. This evidence of overlapping post-response neural activity provides evidence for automatic encoding of confidence independent of any explicit request for metacognitive reports and a shared basis for metacognitive evaluations across sensory modalities.

## Significance Statement

Understanding the neural basis of self-evaluation is an important and active area of research. Here we show that error-related activity patterns following a speeded visual task could be used to predict accuracy in a later auditory judgment. This neural activity further generalized to predict confidence in the later auditory decision. This automatic encoding of self-evaluation that is shared between sensory modalities is of theoretical and practical importance, for identifying a domain-general marker of confidence that can improve our understanding of human decision-making.

## Introduction

People can evaluate the quality of their decisions even in the absence of objective feedback, giving graded judgments of confidence (Henmon, 1911) and binary evaluations of accuracy (Rabbitt, 1968) that can correlate remarkably well with their objective performance. There is growing interest in the cognitive and neural mechanisms underpinning these “metacognitive” self-evaluations (Fleming and Frith, 2014), stimulated by evidence of the key role they play in adaptive behaviors such as information seeking (Desender et al., 2018; Pescetelli et al., 2021) and shared decision-making (Bahrami et al., 2010; Silver et al., 2021).

The present research uses an EEG multivariate decoding approach to address three core questions regarding the mechanisms of metacognition. The first is the degree to which there is common coding of metacognitive evaluations across task domains. Whereas some evidence identifies dissociable correlates of confidence across different tasks, such as perceptual decisions versus memory retrieval (Baird et al., 2013; Fleming et al., 2014), other studies have found similar behavioral (Mazancieux et al., 2020 ) and neural markers (Rouault et al., 2023) associated with confidence in tasks as disparate as perceptual decisions and Sudoku puzzles (Su et al., 2022). Whether there is common encoding of confidence across domains is theoretically important, but also of practical significance given interest in exploiting neural signals of confidence to improve human decision-making (Valeriani et al., 2017). Here we investigated the relatively understudied question of whether there are shared neural correlates across sensory modalities.

The second question we addressed is the relationship between graded judgments of confidence and binary judgments of response accuracy. There is an intuitive inverse relationship between judgments that a decision is correct versus judgments that one has made an error (Yeung and Summerfield, 2012). Consistent with this intuition, well-characterized markers of error detection that are observed in response-locked EEG data, the error-related negativity (ERN) and error positivity (Pe), also vary in amplitude in a graded manner with participants’ ratings of confidence in their decisions (Boldt and Yeung, 2015). However, several studies have documented EEG correlates of confidence in stimulus-locked waveforms (Gherman and Philiastides, 2015; Herding et al., 2019). Some studies have suggested further that postresponse EEG activity may not index confidence despite the observed correlation (Rausch et al., 2020) and that the correlation itself may be a statistical artifact (Feuerriegel and Bode, 2022). These arguments align with the view that confidence is primarily determined by the strength of evidence accumulated to the point a decision is made (Vickers and Packer, 1982; Kepecs and Mainen, 2012), whereas post-decisional processing leading to error detection is largely restricted to tasks with speed pressure that induce “fast guess” errors (Rabbitt, 1968). Here we aimed to provide new evidence for a shared dependence of error detection and confidence judgments on post-response neural activity: we predicted that multivariate EEG activity associated with errors in a speeded (visual) task would generalize to predict graded variations in confidence in correct responses in an unspeeded (auditory) task.

Our final question was the degree to which metacognitive evaluations are an automatic part of decision-making. Although some theories make this claim (Lee et al., 2023), there is little direct evidence of how often thoughts are metacognitive (Jordano and Touron, 2018). Early reports of error-related EEG activity were observed in the absence of explicit performance evaluation (Falkenstein et al., 1990), suggesting that error detection can proceed automatically. However, evidence of “reactivity” (Double and Birney, 2019), such that asking participants to make metacognitive judgments alters their decision-making (Petrusic and Baranski, 2003), suggests otherwise. The ubiquity of mind wandering (Killingsworth and Gilbert, 2010) likewise indicates imperfect and inconsistent self-monitoring. In the present study we test for this automaticity by requiring participants to first perform a visual task with no request for (and, indeed, no mention of) self-evaluations of performance, prior to the auditory task which required rating confidence on each trial. Evidence of overlapping neural activity across the two tasks would provide evidence for automatic encoding of confidence independent of any explicit request for metacognitive reports.

## Materials and Methods

### Participants

Twenty-one participants participated in this experiment (12 female, 20–35 years old, *M*_age_ = 25), all with normal or corrected-to-normal vision. Three additional participants were recruited but excluded from subsequent analysis, for failing to adequately vary confidence ratings (single ratings when correct, *n* = 1) or poor EEG data quality (*n* = 2). All participants gave written informed consent and were paid for their participation. The procedures were approved by the University of Oxford's local ethics committee.

### Task and procedure

**Figure 1.**
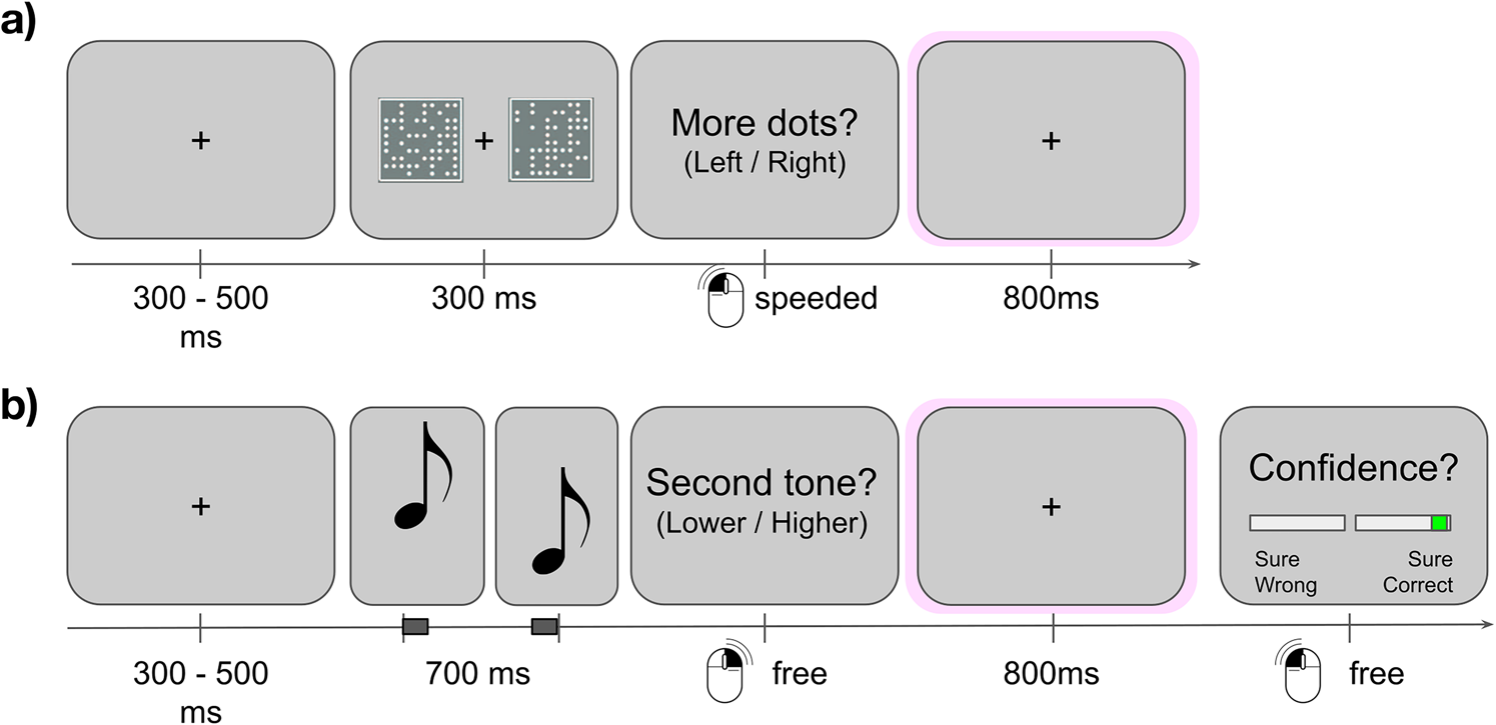
Experimental paradigm. ***a***, In the first half of each experiment, participants completed a speeded visual decision task. Immediately after stimulus offset, participants were prompted to make their decision, before a fixed post-decision window containing only a small fixation cross was included to enable across-session comparison of neural activity. ***b***, In the second half, participants completed an auditory decision task and additionally rated their confidence on each decision.

The experiment was designed for a within-participant comparison of neural activity following visual and auditory perceptual decisions, with subjective confidence ratings only after the auditory task. We were motivated to explore whether post-decision error signals, in the absence of confidence judgments, could generalize to predict confidence formation in a separate modality. To this end, participants performed separate visual and auditory tasks in a fixed order while we recorded EEG data. Participants first completed blocks of a speeded visual discrimination task with no requested performance evaluations, before moving on to blocks of an auditory task for which they reported their subjective confidence after each decision (Fig. 1).

For the visual trials, participants were required to judge which of two square fields presented to the left or right of fixation contained more dots (300 ms duration, 400 dots total, left/right mouse click; Fig. 1). Participants were not prompted to evaluate their trial-wise performance at all during this stage—from their perspective, they simply performed an uninterrupted series of visual discriminations—and no written or verbal mention of confidence was provided to participants until after this phase was completed. The dot difference between fields was titrated per participant to approximate 84% accuracy, using a pre-experimental staircase procedure. During this staircase, three blocks of 30 trials were presented without feedback (one-up four-down adaptive staircase, increment two dots). After the staircase, the dot difference was fixed for the remaining 13 blocks of 30 trials of the visual experiment. If individual block accuracy exceeded 90%, or mean reaction times exceeded 2 s, participants were prompted to try to respond more quickly. Similarly, if block accuracy fell below 70%, participants were instructed to respond more carefully. This emphasis on speed was used to enable the planned contrasts of neural activity on correct versus error trials, ensuring sufficient numbers of errors, and also that many of these would be “fast guess” errors that are typically associated with robust post-response error potentials (Scheffers and Coles, 2000). Importantly, after each response, we included a long intertrial interval (jittered with uniform distribution 1,100–1,300 ms), to facilitate the across-task decoding of response-locked neural signals related to the visual decision.

After completion of the visual portion of the experiment, participants were introduced to the auditory task and the additional confidence judgments that were required after each decision. On each trial in this phase, participants listened to two pure sine-tones in a quick sequence (100 ms tone, 500 ms gap, 100 ms tone) and were asked to judge whether the second tone of the sequence was lower or higher in pitch. The lower pitch was selected between 300 and 350 Hz on each trial. The frequency of the higher-pitch tones was set using an adaptive staircase procedure similar to the visual portion of the experiment, during which the ratio between low and high pitch tones was titrated to approximate 84% accuracy. Three blocks of 30 auditory calibration trials were presented during the staircase. After calibration, a further 10 blocks of 30 trials were completed at the fixed difficulty level. After each response on the auditory task, a fixed 800 ms interval was introduced before participants indicated their confidence in their previous decision. Confidence judgments were provided by clicking one of two slide bars, the first was labeled “Sure wrong” to “Maybe wrong” and ranged from 100 to 50% on the left of fixation, and the second ranged from 50 to 100% “Maybe correct” to “Sure correct” on the right of fixation (Fig. 1). After clicking either slider, participants submitted their response by pressing the space bar. There were no speed prompts between blocks in the auditory portion, confidence responses were not speeded, and after the response, the next trial began after a jittered 300–500 ms interval.

Stimuli were presented on a 17 inch monitor with a 60 Hz refresh rate using MATLAB (version 2020) and Psychtoolbox routines (Brainard and Vision, 1997). Dot fields were presented within a square frame (6°of visual angle wide) centered 5° from fixation (framewidth 0.1°). Each box was subdivided into a 20 × 20 grid, with square dots placed within grid locations selected from a random uniform distribution. Auditory stimuli were played at a comfortable volume over speakers placed either side of the monitor.

### EEG recording

Participants sat in an electrically shielded room and EEG was recorded at 1,024 Hz from 64 active electrodes corresponding to the 10–10 system (actiCAP, Brain Products). An additional six electrodes were placed on the left/right canthi, above/below the right eye, and on the left/right mastoid processes to monitor oculomotor activity and for offline re-referencing. All data was referenced online to FCz and rereferenced to the average of both mastoids during preprocessing. During preprocessing, EEG data were downsampled to 256 Hz, referenced to the average of both mastoids, and filtered between 0.1 and 30 Hz (zero-phase noncausal, 8,449 point order, 0.1 Hz transition bandwidth). EEG were then epoched from −500 to +3,500 ms relative to stimulus and response onsets. These datasets were then merged, and individual epochs inspected by eye for trial rejection. On average, <4% of trials were identified for exclusion per participant. An independent components analysis was performed to identify and remove blinks, oculomotor and other artifacts using the SASICA toolbox (Chaumon et al., 2015). After the ICA, automatic detection of channels with large kurtosis values (*Z* > 5) were spherically interpolated using nearest neighbors (average 2.7 channels per participant).

### Behavioral data analysis

Accuracy and reaction times were calculated for each portion of the experimental task, excluding practice sessions. Visual reaction times were recorded from stimulus onset, and auditory reaction times were calculated from the onset of the second tone stimulus. Confidence judgments were first *z*-scored within each participant to facilitate across-participant comparisons.

### EEG analysis

Event-related potentials were calculated aligned to visual stimulus onset, second auditory tone onset, and response onset per participant. Epochs were first linearly detrended and then baseline corrected. Stimulus-aligned epochs were baseline corrected relative to a 100 ms prestimulus window, and response-locked data were corrected using a −100 to −50 ms window in order to omit the interval containing the error-related negativity (ERN; compare Fig. 3). No statistical method was used to select electrodes for ERP analyses, with subsets selected to visualize each response based on scalp topography and conventions from past research (Boldt and Yeung, 2015). We note that the use of preresponse baseline may bias post-decision ERP amplitudes when systematic differences exist in the preresponse time window (Feuerriegel and Bode, 2022). In our dataset, we are not focused on interpreting the amplitude of the post-response waveform, but whether systematic whole-scalp variability encodes a metacognitive signal that is shared between tasks. This focus notwithstanding, we have also repeated our main analysis using a prestimulus baseline for comparison, and note our main results are not contingent on the preresponse baseline. For our univariate ERP analysis, we calculated ERPs on objectively correct and error trials from both the visual and auditory portions of the experiment (compare Fig. 3), as well as ERPs for the subset of subjectively correct trials when split by either low or high confidence from the auditory portion of the experiment. For this analysis, a median split of confidence values was used, owing to insufficient variation in confidence judgments to split by terciles of quartiles on a subset of participants (5/21).

For our main analysis, we trained a classifier to discriminate between visual task correct and error trials using single-trial response-locked ERP waveforms (Parra et al., 2002; Boldt and Yeung, 2015). We were particularly motivated to test whether information in the Pe window (250–350 ms post-response) would generalize to predict response accuracy and confidence judgments in a separate auditory task. To robustly quantify Pe magnitude on single trials of the visual task, we trained a multivariate classifier to distinguish between the response-locked ERPs generated on error and correct trials using the linear integration method (Parra et al., 2002). This method identifies the spatial topography that maximally discriminates between conditions of interest, which can be interpreted as a vector of weights that combine information from all electrode locations. Similar to conventional ERP analyses which improve signal-to-noise ratio by averaging over many channels, the linear integration method can improve the signal-to-noise ratio of single-trial data by combining the data across all electrodes via their weighted sum. This approach has previously been used to show that single-trial fluctuations in Pe amplitude predict subtle variations in subsequent confidence judgments (Boldt and Yeung, 2015). Here, we address whether a classifier trained in this manner can generalize to another modality and whether single-trial Pe estimates are predictive of auditory decision confidence.

Our first analysis trained classifiers on the 250–350 ms window of post-response activity to align with the visual error-related positivity (Pe; for similar Steinhauser and Yeung, 2010; Boldt and Yeung, 2015). First, at the participant level, classifiers were trained using all error trials (Boldt and Yeung, 2015) and a matched size subset of correct trials which were selected at random from a uniform distribution (without replacement). As the selection of training trials could, in principle, have derived a different classifier, we repeated the selection of training trials over iterations of 5, 10, 20, and 50 validations. We note that participant-level results were stable when using five or more iterations and report results after calculating participant-level effects using 20 iterations. At the participant level, classifier performance was assessed by quantifying the proportion of trials that were correctly classified as errors or correct responses. Figure 4 displays the group-level data after averaging across these participant-level effects. We display the probability of being labeled an error separately for both correct and error trials in the visual and auditory portion of the experiment, as well as the area under the ROC curve (AUC) when tested on all untrained trials.

We also examined the temporal generalization of classifier accuracy (King and Dehaene, 2014) and extended our training window and data types to include all timepoints −500 to 1,000 ms relative to both stimulus-onset and response-onset locked ERP waveforms. This analysis was performed to visualize whether error and correct trials in an auditory task could also be distinguished based on any stimulus-locked activity and whether activity in the response-locked Pe window was unique in this regard. This analysis used a sliding window approach creating classifiers based on 50 ms windows of activity progressing in steps of 25 ms. Classifier training and test data were selected as above. The temporal generalization of each classifier was assessed by applying the topography obtained from each 50 ms window to all timepoints, before averaging AUC values within each 50 ms training window for analysis and visualization (Fig. 5). As classifiers were trained and tested on all timepoints, this creates a 2D performance matrix.

Contiguous significant clusters of activity were first identified at an alpha level of *p* < 0.05 (two-tailed, uncorrected, *t* test against chance), and the absolute sum of *t* values was retained per cluster for comparison with a null distribution of cluster-level test statistics. To create the null distribution, the temporal generalization of classifier performance was repeated on trials with randomized condition labels (1,000 permutations). The maximum cluster-level test statistic was retained per permutation, as described above, and the originally observed cluster-level statistics were considered significant when exceeding the bottom 95th percentile of this null distribution (e.g., *p*_cluster_ < 0.05). The results of this analysis are displayed in Figure 5.

Finally, after demonstrating that the Pe window classifier could generalize to a cross-modal task, we investigated whether classifier output would correlate with single-trial confidence ratings. For this analysis, we restricted our focus to only include subjectively correct trials—trials in which participants rated their confidence between 50 and 100% “Sure Correct.” This subset excludes trials in which participants may have changed their mind after their initial response and detected errors which may otherwise confound overall classifier performance: Here we are interested in gradations of correct-trial confidence as distinct from their ability to detect their errors. Specifically, we applied the classifier weights derived from the visual Pe window to all auditory response-locked data using a sliding window approach (50 ms window, 5 ms step size), retaining a score per timepoint across all trials for evidence of an objectively correct or error response (Bernoulli probability distribution, ranging from 0 to 1, respectively). All trials were then correlated with their respective confidence ratings, and the correlation visualized per 5 ms step in our sliding window approach. This analysis was first performed at the participant level, producing a time-series for the correlation between classifier performance and subjective confidence, before averaging across participants. The group-level result effectively quantifies whether (and when) confidence judgments correlate with neural activity that resembles visual error detection during the auditory portion of the task. The results of these analyses are shown in Figure 6. As above, we corrected for multiple comparisons in the correlation time-series using a nonparametric cluster-based correction (Maris and Oostenveld, 2007). Temporally contiguous clusters were first detected (at *p* < 0.05, two-tailed, uncorrected) before the summed cluster-level test statistic was retained for comparison to the null distribution. The null distribution was created by shuffling condition labels 2,000 times, before retaining the maximum sum of cluster-level test statistics of each permutation. We regarded the observed cluster-level test statistic to be significant if it exceeded the bottom 95% of the null distribution of the summed statistics (e.g., *p*_cluster_ < 0.05).

## Results

### Behavioral data

**Figure 2.**
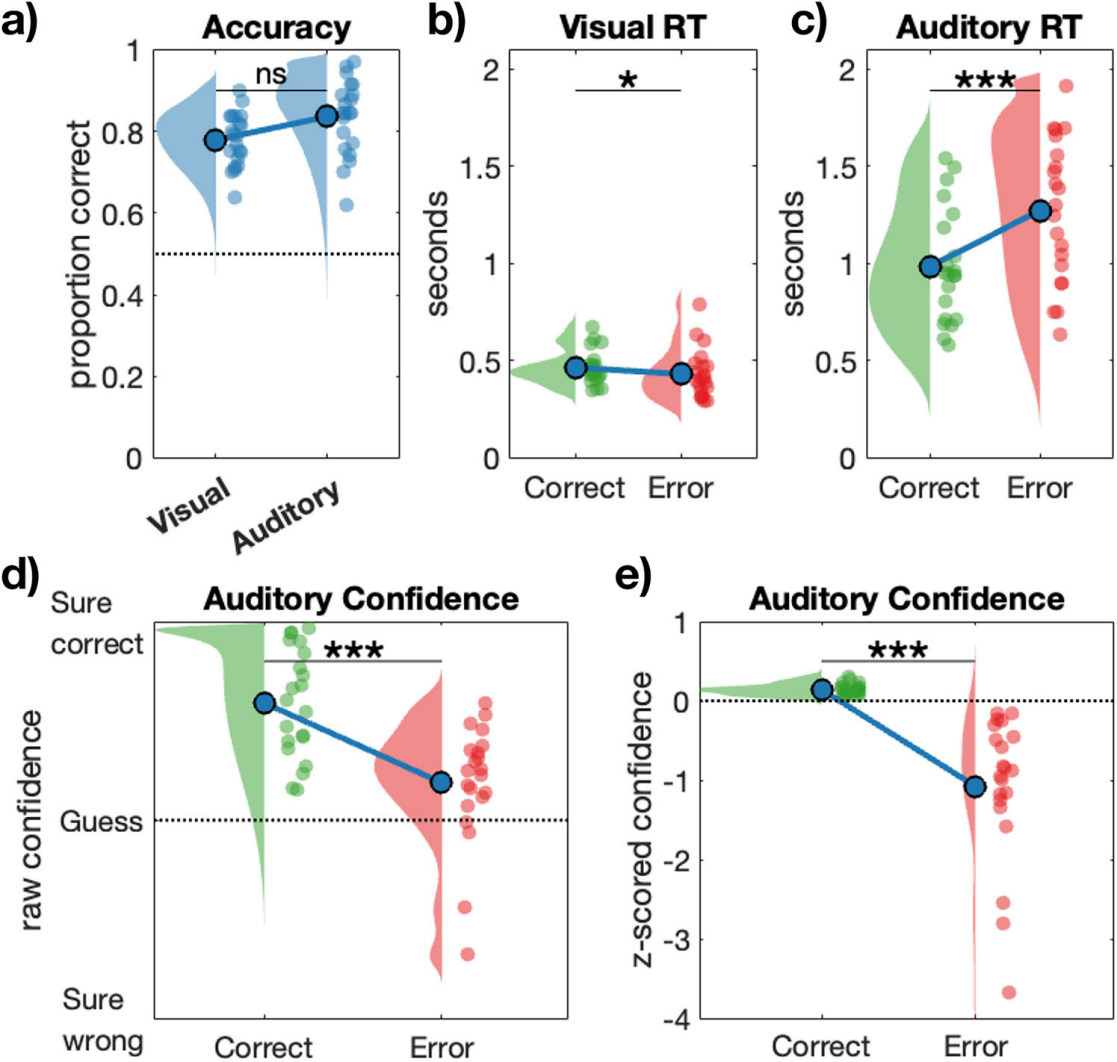
Behavioral results summary. ***a***, Accuracy did not significantly differ between the visual and auditory tasks. ***b***, Reaction times were faster when making errors on the speeded visual decision task, and ***c***, reaction times were slower when committing errors on the auditory decision task. ***d***, Raw confidence values for objectively correct and error trials in the auditory task. Confidence was higher for correct responses than errors, but on average error confidence was in the range “Guess” to “Sure correct”, indicating that participants struggled to detect their errors. ***e***, Confidence (*z*-scored within participants) was calibrated with objective accuracy, with lower confidence overall on incorrect auditory decisions.

Participants responded to a speeded visual perception task indicating which of two locations contained a larger number of dots, as well as an auditory decision task which additionally prompted unspeeded confidence judgments. Figure 1 displays a schematic of the trial structure, and Figure 2 shows the behavioral results.

Both the visual and auditory portions of the experiment began with an adaptive staircase procedure to calibrate performance to ∼84% accuracy. As a result, there was no significant difference in accuracy on the two tasks (*p* = 0.06). However, the relative speed of correct versus error responses differed across tasks. Thus, as is typically observed in speeded tasks with many fast guess errors, visual task reaction times were shorter on error trials (*M* = 0.43 s, *SD* = 0.12) than they were on correct trials (*M* = 0.46 s, *SD* = 0.08; *t*_(20)_ = 2.70, *p* = 0.014, *d* = 0.59). In contrast, when performing the unspeeded auditory portion of the experiment, reaction times were longer on error trials (Errors *M* = 1.27 s, *SD* = 0.37; Corrects *M* = 0.99 s, *SD* = 0.29; *t*_(20)_ = −6.18, *p* < 0.001, *d* = −1.35). Confidence judgments were also higher on correct trials (*M* = 82.45, *SD* = 14.94) than they were on errors (*M* = 60.36, *SD* = 16.56; *t*_(20)_ = 4.38, *p* < 0.001, *d* = 0.96) in the unspeeded auditory task. Despite this difference, participants were predominantly unable to detect their errors in this task, with confidence ratings in the range “Guess” to “Sure correct” on 77% of error trials (*SD* = 22.8; range, 9–98%). Participants’ failure to detect their errors reflects that incorrect responses in the unspeeded auditory task tended to be “data limited errors” related to the difficulty of the perceptual discrimination rather than the occurrence of fast guesses (Scheffers and Coles, 2000) and is a feature we return to in our decoding and generalization analyses.

### Univariate ERP data

**Figure 3.**
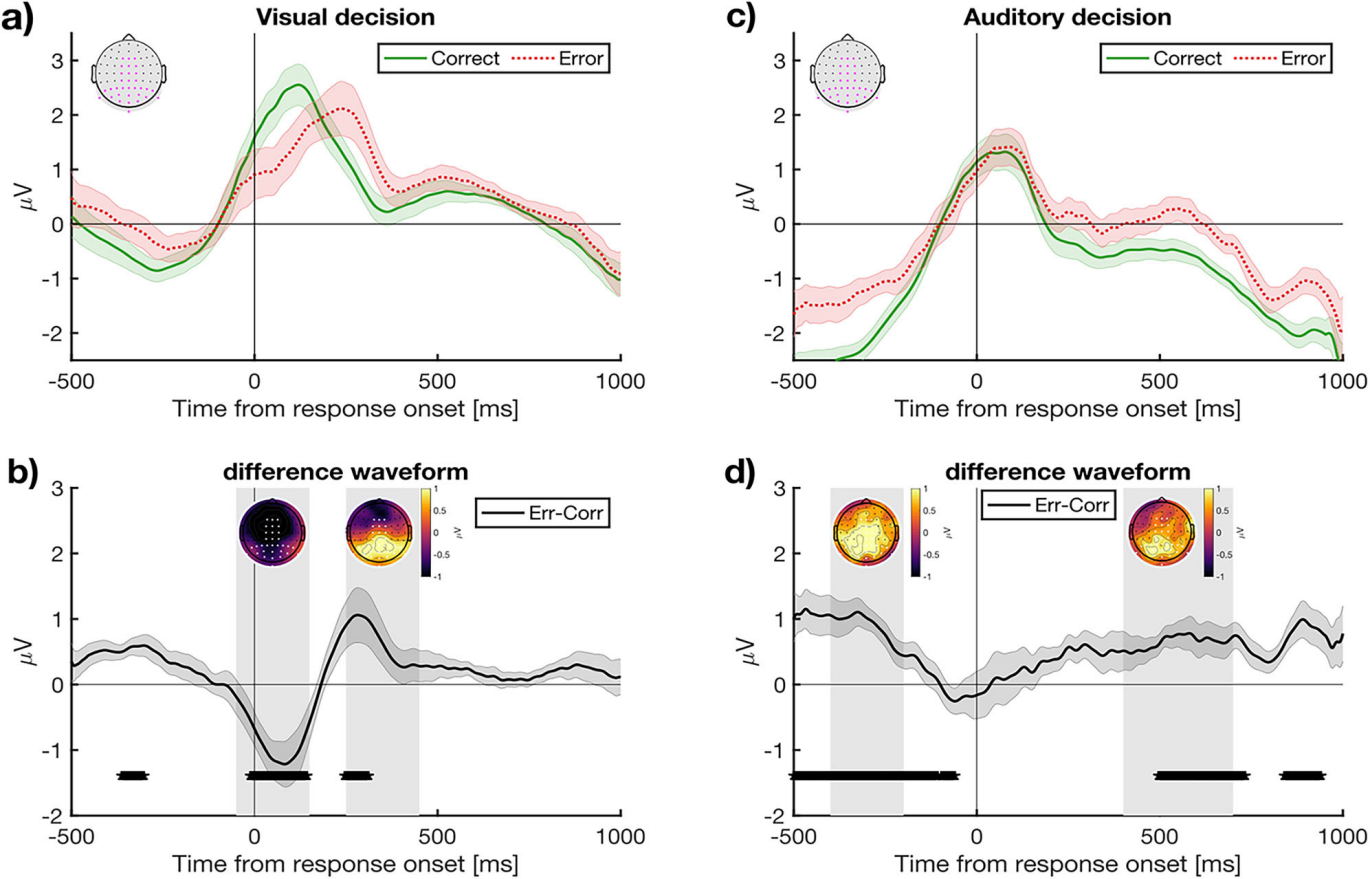
Response-locked ERPs following a visual and auditory decision. ***a***, ERPs time locked to responses on the visual task, split by objective accuracy showing correct (green) and error (red) trials. ***b***, ERPs time-locked to responses in the auditory task. In all panels, the ERPs are constructed from the electrodes marked in ***a***–***b*** in magenta. ***c***, The difference waveform (error − correct) for visual responses. The inset plots show scalp topography for the average activity over the two temporal windows shaded in gray (−50 to 150 ms and 250–450 ms). ***d***, Difference waveform for auditory responses, with inset plots showing the scalp topography for the two temporal windows (−400 to −200 ms and 400–700 ms). Horizontal bars indicate *p* < 0.05 (uncorrected). Shaded error bars display the standard error of the mean corrected for within-participant comparisons (Cousineau, 2005).

We proceeded by investigating the post-decision event-related potentials. Figure 3 displays a summary of the response-locked univariate ERP data. Consistent with prior research, when computing the difference waveform between error and correct trials in the visual task, we observed a pronounced fronto-central negativity beginning around the time of the response and peaking shortly after, followed by a positive component ∼200–400 ms post-response that was maximal over posterior scalp sites. These components align with the error-related negativity (ERN), and error positivity (Pe), implicated in previous investigations of post-decision confidence formation. The amplitude of the Pe in particular has been shown to correlate with subjectively reported confidence (Boldt and Yeung, 2015), and whether neural activity in this window generalizes to predict auditory decision-making is our main focus. For comparison, the right-hand panels of Figure 3 plot the corresponding waveforms for the auditory task. There is no prominent ERN component, but a Pe is apparent as an increased positive voltage for the error-trial waveform over posterior sites. Compared with the visual task Pe, however, the auditory task Pe has a markedly slower onset and longer duration.

### Single-trial multivariate decoding

Our univariate analysis revealed pronounced differences in ERP activity when comparing correct and error trials in both the visual and auditory tasks. Notably, the magnitude and morphology of these responses differed based on the modality of the task (Fig. 3*c*,*d*). However, of key interest was whether we would nevertheless find evidence of shared neural signatures of metacognitive monitoring in the two tasks. To this end, our next analysis investigated whether a multivariate classifier trained to predict error responses in the visual task would generalize to correctly distinguish errors and correct responses in an auditory decision.

**Figure 4.**
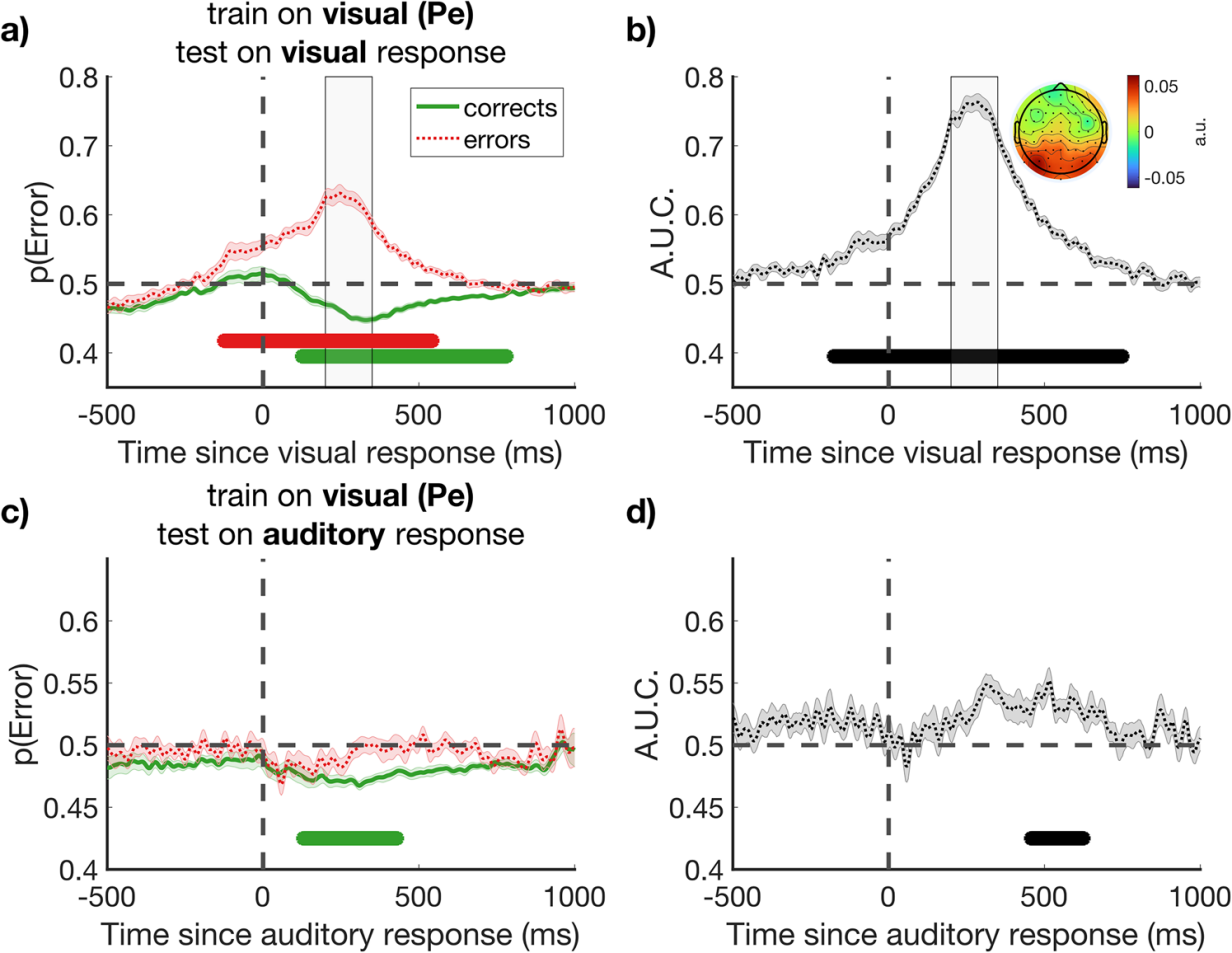
Temporal and cross-modal generalization of a classifier trained to predict errors on a visual perceptual decision. ***a***, Temporal generalization of classifier performance when trained on the Pe window (250–350 ms, window shown in gray in ***a***, ***b***). Classifier performance significantly differs from chance on both error and correct trials (red and green horizontal bars, respectively, *p*_cluster_ < 0.001), ***b***, with high classification performance overall (black horizontal bars *p*_cluster_ < 0.001). The group-level weights assigned to each electrode are shown in the inset topoplot (arbitrary units). ***c***, The same classifier (trained on visual task data) generalizes to predict correct trials on a later unspeeded auditory decision. ***d***, Significant above-chance classification occurs in the post-response window (*p*_cluster_ < 0.01). Shaded error bars display the standard error of the mean corrected for within-participant comparisons (Cousineau, 2005).

For this analysis, we focused on the Pe in a window from 250 to 350 ms post-response, in line with our previous work (Steinhauser and Yeung, 2010; Boldt and Yeung, 2015). We trained our classifier to distinguish error and correct trials of the visual task to derive a spatial weighting of electrodes that, when convolved with observed scalp voltages, maximally distinguishes those trial subsets. The resulting weighting can then be applied to other data outside the training set, with the output being a value for each timepoint that corresponds to the estimated probability that the trial belongs to the target category (here: errors). The temporal generalization of the classifier as applied to the response-locked EEG data from the visual task is shown in Figure 4*a*, for correct and error trials separately (corrects: *p*_cluster_ < 0.001, 125 to 781 ms post-response; errors: *p*_cluster_ < 0.001, −125 to 543 ms post-response). The classifier shows significant classification of both errors and correct responses for sustained periods in the post-response period, assigning the former a p(Error) value >0.5 and the latter a value <0.5. When tested on all trials, the area under the ROC curve is displayed in Figure 4*b*. Peak classification accuracy occurs within the training window, as expected, but above-chance decoding accuracy is also clear for earlier and later periods around the time of response (*p*_cluster_ < 0.001, −175 to 750 ms post-response).

Importantly, we next tested the visual classifier on the auditory response-locked data, wherein none of the auditory trials were used in training, and observed significant above-chance classification when tested on all trials over the period 457 to 625 ms after the response (area under ROC curve, *p*_cluster_ < 0.001; Fig. 4*d*). These findings suggest overlapping post-decisional neural markers of metacognition across the visual and auditory tasks. The correspondence between the timing of significant cross-classification (Fig. 4*d*) and of the auditory task Pe (Fig. 3*b*,*d*) is consistent with this component being the shared neural correlate of metacognitive evaluation. The time-limited nature of cross-classification, which dissipates toward the end of the response-locked epoch, rules out the results being caused by stimulus-locked activity contaminating the response-locked baseline period we use (cf. Feuerriegel and Bode, 2022), any effect of which should persist throughout the epoch. Confirming this, similar results are observed when we reanalyze the data using a prestimulus rather than preresponse baseline. Notably, classification primarily reflected assignment of P(Error) values lower than 0.5 to correct auditory task trials (corrects: *p*_cluster_ < 0.01, 128 to 430 ms post-response), rather than values above 0.5 to errors (Fig. 4*c*). For errors, the P(Error) classifier value did not significantly exceed 0.5 at any timepoint. This result is partially to be expected given that participants failed to detect the majority of their errors in the auditory task (compare Fig. 2*d*) and is a first indication that the classifier is generalizing to identify variations in confidence in correct responses, rather than only participants detecting their errors, a theme explored further below.

### Temporal generalization of cross-modal classifier accuracy

Figure 4 demonstrates that a classifier trained on the Pe window following a visual decision can generalize to classify response accuracy in a later, unspeeded auditory decision. The time-course of changing classifier accuracy which extends beyond the training window (compare Fig. 4*b*,*d*) also demonstrates the temporal generalization of this cross-modal classification. Our next analysis formally quantified the extent of this temporal generalization by comparing all stimulus-locked and response-locked timepoints, using the temporal generalization method (King and Dehaene, 2014).

**Figure 5.**
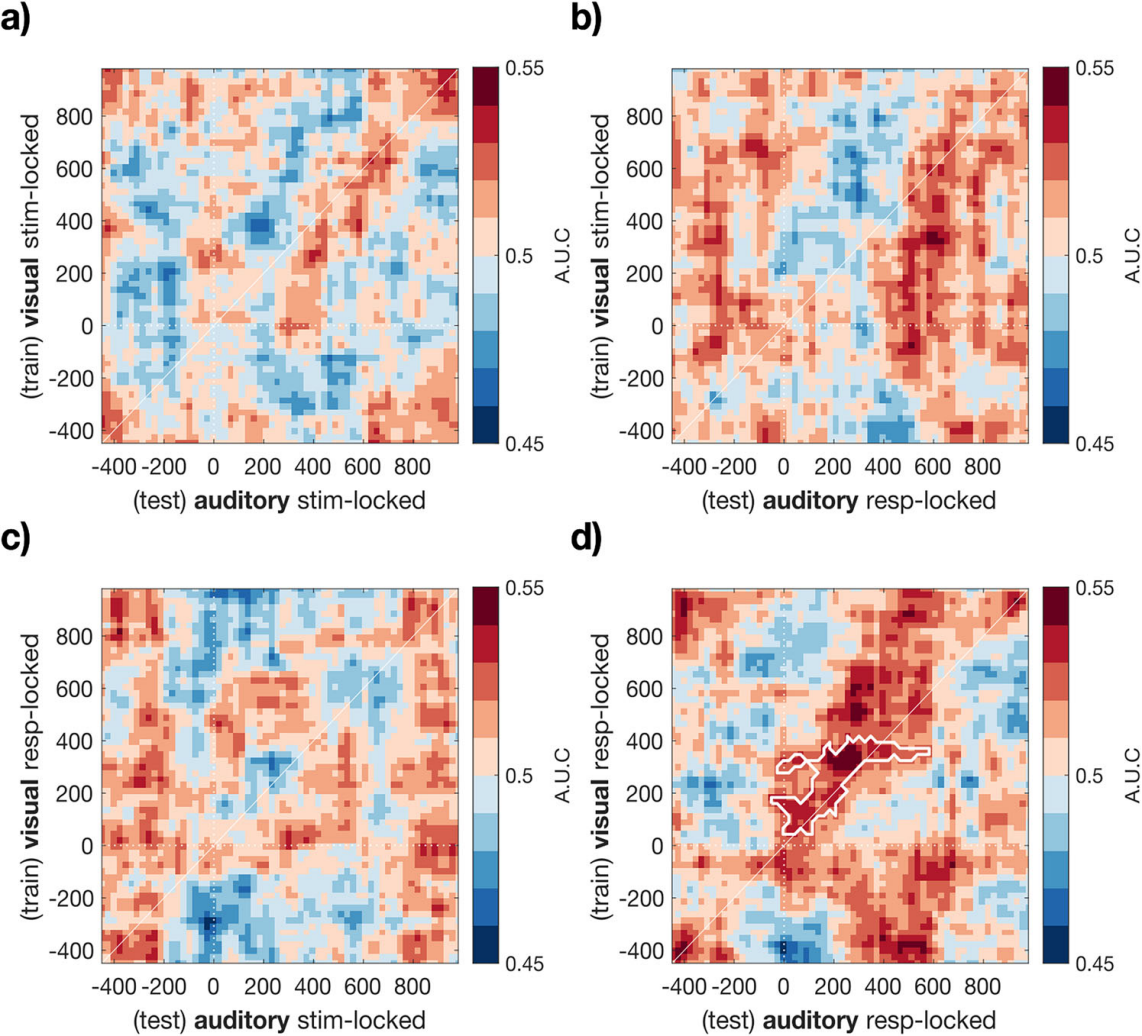
Cross-modal and temporal generalization of error classification. Each panel shows the temporal generalization matrix of multivariate classifiers trained on either the visual stimulus (***a***, ***b***) or visual response-locked ERP (***c***, ***d***) and tested on either the auditory stimulus-locked (***a***, ***c***) or response-locked ERP (***b***, ***d***). Only a classifier trained on the visual response-locked ERP data generalizes to predict errors in a subsequent auditory task (***d***; white lines denote boundaries, *p*_cluster_ < 0.001 corrected for multiple comparisons; Maris and Oostenveld, 2007).

For this analysis, we repeated the same procedure as described above but now using a variety of time windows in both stimulus- and response-locked epochs for both training and testing. With this approach, we tested whether the generalized prediction of cross-modal error was unique to the response-locked Pe window, or would also be observed in earlier stimulus-locked differences in ERP amplitude, and further whether cross-classification would be possible between stimulus- and response-locked waveforms. Figure 5 shows the results of this analysis. Classifiers trained on stimulus-locked ERP waveforms from the visual task did not lead to above-chance decoding accuracy for auditory decisions, either when tested on stimulus-locked (Fig. 5*a*) or response-locked waveforms (Fig. 5*b*). Nor did classifiers trained on visual task response-locked waveforms generalize to classify auditory task trials based on stimulus-locked EEG activity (Fig. 5*c*). Significant cross-classification was only apparent in the response-locked waveforms (Fig. 5*d*). This result extends the basic classification analysis reported above (Fig. 4) to show that significant cross-task classification is evident for a range of training and testing timepoints across the window from 0 to 600 ms post-response. Cross-classification is primarily observed along the diagonal. The limited temporal generalization (i.e., classifiers trained on one timepoint do not robustly cross-classify data from distant timepoints) can be indicative of a dynamic sequence of neural representations (King and Dehaene, 2014).

### Classifier scores correlate with single-trial confidence ratings

**Figure 6.**
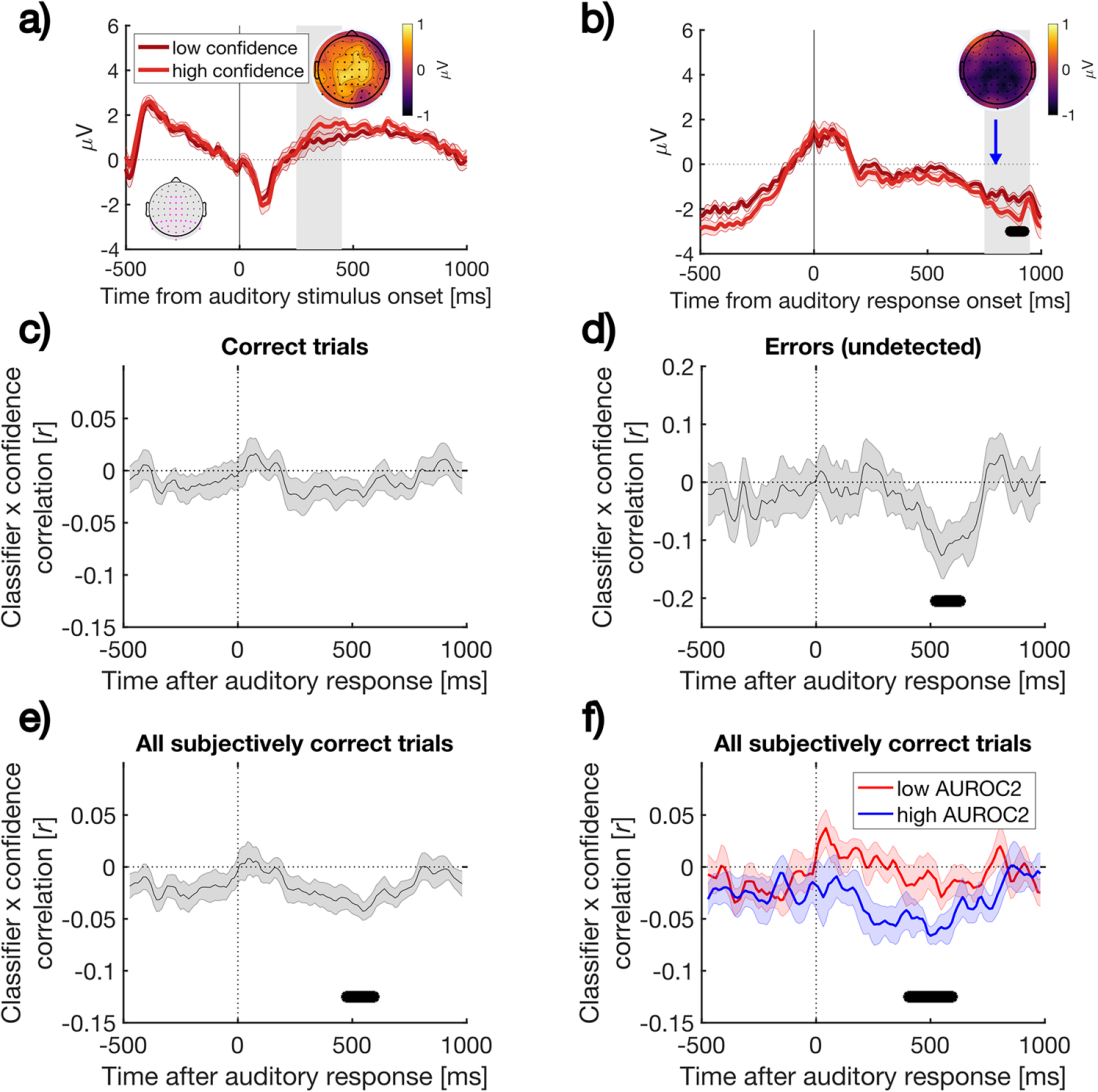
Visual error detection underpins auditory confidence. ***a***, ***b***, Low- and high-confidence ERP waveforms defined by median split per participant, within the restricted subset of subjectively correct trials only. ***a***, auditory stimulus-locked waveforms; ***b*** auditory response-locked waveforms. Channels used to compute ERPs are shown in the inset (gray, magenta). Topoplots of average activity within gray shaded windows of interest 250–450 ms and 750–950 ms poststimulus and response, respectively, are included. Topoplots show the difference between low and high confidence trials. The vertical blue line indicates the onset of the confidence response screen. ***c***, Single-trial correlation between error classification and confidence judgments (see Materials and Methods). The topographic weights from a classifier trained exclusively on the Pe window following responses to a visual stimulus were applied to the activity in all auditory response-locked waveforms using a sliding window approach, restricting to subjectively correct trials only. The classifier linearly integrates activity over all electrodes to produce a probability for the likelihood of a visual error. ***c***, The classifier did not correlate with confidence on objectively correct trials. The negative correlation in ***d*** indicates that confidence judgments on the auditory task decrease as auditory response-locked neural activity resembles visual errors, even when participants were unaware of these errors and rated confidence in the range of subjectively correct. ***e***, The result from all subjectively correct trials. ***f***, The same result as ***e*** after median split of the participants based on type-2 AUC (metacognitive sensitivity). Horizontal bars indicate *p*_cluster_ < 0.05. Shaded error bars display the standard error of the mean corrected for within-participant comparisons (Cousineau, 2005).

Our final analysis provided the critical evaluation of whether our classifier trained to distinguish correct from error responses in a visual task would generalize further to predict subtle fluctuations in confidence judgments in an auditory task. We hypothesized that as well as showing above-chance decoding accuracy (cf. Fig. 4*d*), classifier scores would also negatively correlate with confidence judgments in a time-dependent manner [negatively because the classifier output corresponds to P(Error), which should vary inversely with participants’ estimates that their responses are correct]. Evidence consistent with this prediction would demonstrate that a supramodal error signal formed in the absence of explicit confidence reports also underpins the strength of later confidence judgments in a cross-modal task. For this analysis, we applied the classifier trained on the visual Pe window to all timepoints within the auditory response-locked data and calculated the correlation using a sliding window approach. We were specifically interested in variations in participants’ confidence in correct decisions and therefore restricted analysis to trials in which participants rated their confidence as above 50% (i.e., excluding trials on which participants detected errors). Figure 6 displays a summary of the results.

Figure 6 plots ERP waveforms when subjectively correct trials are median split into low versus high confidence subsets and the data are aligned to stimulus or response onset. The waveforms show only modest differences, with small and nonsignificant differences in the time range of the Pe (where we might expect lower confidence trials to be associated with a Pe-like positivity). Small differences in ERP amplitude within subjectively correct trials emerged close to the time of providing a subjective confidence judgment. Nevertheless, as predicted, when applied to these subjectively correct trials, the scalp topography used to distinguish error trials in the visual portion of the experiment negatively correlated with confidence judgments in the auditory portion of the task (Fig. 6*d*,*e*). Thus, the EEG-derived estimate of P(Error) for the time window from ∼500–600 ms after the response predicted participants subsequently reported confidence. As with cross-classification of auditory task accuracy (Fig. 4*d*), the time-course of this correlation tracked the morphology of the Pe component. Ruling out that the correlation reflected contamination of our response-locked baseline by stimulus-locked activity, the correlation was clearly time limited rather than sustained to the end of the analyzed epoch. We note that the correlation did not reach significance when applied to all objectively correct trials (Fig. 6*c*) as it did on all subjectively correct trials (Fig. 6*f*). However, there were lower trial counts to quantify this correlation for objectively correct trials (*M* = 250.81, SD = 27.15), than subjectively correct trials (*M* = 281.33, SD = 22.69) as the latter could also include (undetected) objective errors.

As further evidence that the classifier was sensitive to a shared neural marker of error detection and subjective confidence, we repeated the analysis in Figure 6*e* after performing a median split of our participant sample based on metacognitive sensitivity. We quantified metacognitive sensitivity (type 2 performance) as the area under the receiver operating characteristic curve per participant, which captures the fidelity of confidence judgments to predict objective performance. Figure 6*f* displays that the group-level result of a negative correlation between classifier performance and confidence values is primarily driven by the subset of participants with relatively higher metacognitive sensitivity, whose confidence values were superior indicators of objective accuracy.

## Discussion

Our EEG decoding and generalization analyses provide evidence for shared neural correlates of implicit error detection in a visual task and explicit confidence judgments in auditory decisions. We trained a multivariate classifier to distinguish the EEG activity observed following errors versus correct responses in a visual perceptual task. We found, as predicted, that this classifier generalized to distinguish correct responses from errors in a separate auditory pitch-comparison task and further to predict variations in participants’ reported decision confidence in that task.

A first implication of our results is that there are at least partially overlapping markers of metacognitive evaluations across tasks involving different sensory modalities. This finding adds to a growing corpus of neuroimaging evidence for common coding of confidence across different kinds of decisions (Fernandez-Vargas et al., 2021; Su et al., 2022; Rouault et al., 2023), a finding that has also been reported in other species (Masset et al., 2020). This evidence notwithstanding, several studies have also reported dissociations, for example, between neural mechanisms supporting metacognitive evaluations in perception and memory (Baird et al., 2013; Fleming et al., 2014). Indeed, evidence of both overlap and dissociations have sometimes been reported in the same study (Morales et al., 2018; Qiu et al., 2018; Su et al., 2022), leading to the idea of a hierarchical structure whereby domain-general confidence signals in frontoparietal circuits are transformed into content-rich, task-dependent representations in anterior prefrontal cortex that subserve adaptive, context-specific control of behavior. Within this framework, the purpose of domain-general confidence representations might lie in allowing generalization of adaptive behaviors (such as information seeking when unsure) across different cognitive tasks (Kornell et al., 2007; Desender et al., 2018), as well as in allowing adjudication between different kinds of decisions based on their relative reliability (de Gardelle and Mamassian, 2014). Meanwhile, there is practical value in identifying domain-general markers of confidence given their potential use in improving human decision-making via brain–computer interfaces (Valeriani et al., 2017). Our contribution is to demonstrate this domain-generality using EEG decoding across tasks in different sensory modalities.

Our second, related contribution is to locate a shared basis of metacognitive evaluations in post-decisional processes. Our multivariate decoding analysis focused on the time window of the response-locked Pe component, which has previously been shown to vary with graded evaluations of error likelihood (Steinhauser and Yeung, 2010) and confidence (Boldt and Yeung, 2015) in the visual task used here. Error detection inherently depends on continued processing after an initial decision that leads to a change of mind (Rabbitt, 1968). The sensitivity of confidence judgments to post-decisional processing is more controversial: Although proposed by some theories (Pleskac and Busemeyer, 2010; Calder-Travis et al., 2024) and supported by various lines of evidence (Yu et al., 2015; van den Berg et al., 2016; Charles and Yeung, 2019), counter-arguments and counter-evidence exist (Rausch et al., 2020; Feuerriegel et al., 2022). Here we show that the association between post-decisional EEG activity and confidence is not restricted to speeded tasks with frequent “fast guess” errors: Our auditory task was unspeeded and its errors were typically slow responses. Nor can the correlation we observe between Pe amplitude and confidence be explained in terms of artifacts arising from stimulus-locked activity contaminating a response-locked baseline (Feuerriegel and Bode, 2022) because, as also shown previously (Boldt and Yeung, 2015), the relationship is time limited and holds even in analyses using a prestimulus baseline. Indeed, we found reliable cross-task decoding only in response-locked data, with no significant generalized decoding in analyses of stimulus-locked data. The lack of stimulus-locked decoding of confidence is initially surprising given longstanding evidence linking confidence to the amplitude of the stimulus-locked P3 component (Hillyard et al., 1971; Gherman and Philiastides, 2015; Herding et al., 2019 ). However, our approach may not be optimal for exploring the confidence–P3 relationship, for which an approach based on decoding confidence directly (rather than indirectly via initially decoding the contrast between errors and correct trials) might produce more positive results. That is a potential direction for future research. Our contribution here is to demonstrate overlap between neural correlates of error monitoring and confidence judgments via a shared dependence, across tasks, on post-decisional processing.

The third question addressed in our study is the degree to which metacognitive evaluations are an automatic and inherent part of the decision-making process. Although often assumed, and sometimes explicitly theorized (Lee et al., 2023), there is little direct study of how often thoughts are metacognitive (Jordano and Touron, 2018). Error-related EEG activity was originally reported in tasks that did not ask participants to make any explicit performance evaluation (Falkenstein et al., 1990) and it shows only small modulation when explicit reports are requested (Grützmann et al., 2014), suggesting that error detection proceeds automatically and implicitly in typical cognitive tasks. Here we have shown that error-related signals observed in a visual task with no explicit performance monitoring component generalize to predict participants’ confidence reports in a separate auditory task. To the degree that these findings indicate that the Pe component is best characterized as a graded signal that reflects subtle variation in confidence, not simply a binary classification of decisions as correct versus incorrect, they suggest that people generate graded evaluations of confidence even when there is no requirement for them to do so.

Future research may wish to leverage this evidence of a shared domain-general metacognitive signal, yet critical questions remain. While we have focused on two nominally distinct tasks (visual speeded vs auditory unspeeded), both required a binary two alternative forced-choice response. Extensions beyond binary tasks, which also vary response protocols, could provide further evidence of an automatic and generalizable metacognitive self-evaluation signal.

## Synthesis

Reviewing Editor: James Howard, Brandeis University Decisions are customarily a result of the Reviewing Editor and the peer reviewers coming together and discussing their recommendations until a consensus is reached. When revisions are invited, a fact-based synthesis statement explaining their decision and outlining what is needed to prepare a revision will be listed below. The following reviewer(s) agreed to reveal their identity: Simon Kelly. Note: If this manuscript was transferred from JNeurosci and a decision was made to accept the manuscript without peer review, a brief statement to this effect will instead be what is listed below.

This paper advances the understanding of metacognitive processes by showing that, in a speeded visual task with no requirement for metacognitive reporting, neural signatures distinguishing errors from correct decisions significantly predict the level of confidence in correct decisions in a separate, un-speeded, auditory task. These findings suggest the operation of a supra-modal, automatic error signal. The reviewers believe the paper makes a rigorous and interesting contribution to the area of metacognitive processes. The authors have provided detailed, clear responses to all comments and suggestions, and made appropriate revisions to the manuscript to make all necessary clarifications. While there are no outstanding issues to be resolved, there are a couple of small additional recommendations made with regards to the wording in the Abstract and Significance statement, which may benefit from clearer messaging:

1. The term "response-locked" could be understood as referring to activity either before or after the response. Something like "pre-/post-response" might work better here for instant clarity.

2. The use of "neural activity" in the following sentence may be oversimplified and potentially misleading:

"[...] neural activity following speeded responses in a visual task can predict accuracy in a later auditory judgment."

Please consider something a bit more descriptive. For example, "[...] error-related activity patterns derived from a visual task could be used to predict accuracy [...]".
